# Palaeoenvironment and Its Control on the Formation of Miocene Marine Source Rocks in the Qiongdongnan Basin, Northern South China Sea

**DOI:** 10.1155/2014/240415

**Published:** 2014-10-21

**Authors:** Wenhao Li, Zhihuan Zhang, Weiming Wang, Shuangfang Lu, Youchuan Li, Ning Fu

**Affiliations:** ^1^Research Institute of Unconventional Petroleum and Renewable Energy, China University of Petroleum, Qingdao 266580, China; ^2^School of Geosciences, China University of Petroleum, Qingdao 266580, China; ^3^State Key Laboratory of Petroleum Resource and Prospecting, China University of Petroleum, Beijing 102249, China; ^4^China National Offshore Oil Corporation, Beijing 100027, China

## Abstract

The main factors of the developmental environment of marine source rocks in continental margin basins have their specificality. This realization, in return, has led to the recognition that the developmental environment and pattern of marine source rocks, especially for the source rocks in continental margin basins, are still controversial or poorly understood. Through the analysis of the trace elements and maceral data, the developmental environment of Miocene marine source rocks in the Qiongdongnan Basin is reconstructed, and the developmental patterns of the Miocene marine source rocks are established. This paper attempts to reveal the hydrocarbon potential of the Miocene marine source rocks in different environment and speculate the quality of source rocks in bathyal region of the continental slope without exploratory well. Our results highlight the palaeoenvironment and its control on the formation of Miocene marine source rocks in the Qiongdongnan Basin of the northern South China Sea and speculate the hydrocarbon potential of the source rocks in the bathyal region. This study provides a window for better understanding the main factors influencing the marine source rocks in the continental margin basins, including productivity, preservation conditions, and the input of terrestrial organic matter.

## 1. Introduction

The quality of marine source rocks depends on the abundance of organic matter (OM) supply, and the OM preservation conditions, and these factors are controlled by factors such as paleoclimate, biologic productivity, redox conditions, sedimentation rate, origin of organic matter, and paleostructure. Among them, biologic productivity and redox conditions are considered to be the most noteworthy [1, 2]. The mechanism of OM accumulation or the developmental patterns of marine source rocks have been under heated dispute ever since the 1980s. Two theories that are “preservation” and “production” have been dominating the argument. The former suggests that OM accumulation is mainly influenced by redox conditions rather than biologic productivity [3, 4]. The latter argues that the biologic productivity contributes more to OM accumulation than preservation [5]. However, a single parameter is unable to describe the mechanism of high-quality marine source rocks. Besides, the fact that anoxic environment is usually inseparable from high productivity of biomass also proved the insufficiency of the theories. Anoxic water masses used to be observed in upwelling regions with high biologic productivity in open continental shelf, such as the coastal upwellings in Peru [6] and Namibia [7] regions.

Marine source rocks developed in continental margin basins are also influenced by the input of terrestrial organic matter (TOM), besides paleoproductivity and redox conditions. Taking West African rift basins (including Niger Basin, Gabon Basin, lower Congo Basin, Angola Basin, and Namibia Basin), for example, they are all located below the estuary of big rivers. Under the overall regression in Miocene, those rivers transported a large amount of eroded materials from African shield to Atlantic edge and formed constructive delta in estuaries, which provided abundant TOM for forming high-quality source rocks.

Compared with them, it lacks large rivers and deltas, but TOM still has certain contribution to Miocene marine source rocks in shallow region of the Qiongdongnan Basin. Hao et al. [8] considered that Neogene marine source rocks have poor hydrocarbon potential because of the insufficient input of TOM, while Li et al. [9] believed that high-quality source rocks can be formed in the deep water area of the Qiongdongnan Basin owing to the relatively higher productivity. This study is aimed to discuss the main controlling factors and depositional patterns of the Miocene marine source rocks in the Qiongdongnan Basin, based on analysis of supply of terrestrial plants, paleoproductivity, and redox conditions.

## 2. Geological Setting

**Figure 1:**
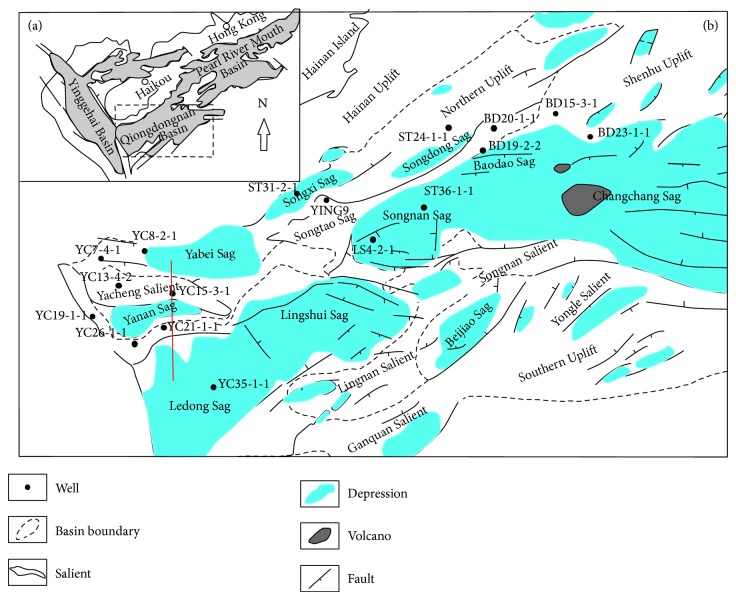
(a) The structural location of the Qiongdongnan Basin [46], northern South China Sea. (b) Geologic map showing the distribution of structural belts in the Qiongdongnan Basin.

Qiongdongnan Basin lies to the southeast of Hainan Island in the northwest of northern South China Sea continental margin. It is composed of several structural zones that are the northern depression belt (including Yabei Sag, Songxi Sag, and Songdong Sag), the middle uplift belt (including Yacheng-Songtao Uplift), the central depression belt (including Lingshui Sag, Ledong Sag, Songnan Sag, and Baodao Sag), and the southern uplift belt (Figure 1). Basin evolution and fill share many similarities with passive margins [10, 11].

**Figure 2:**
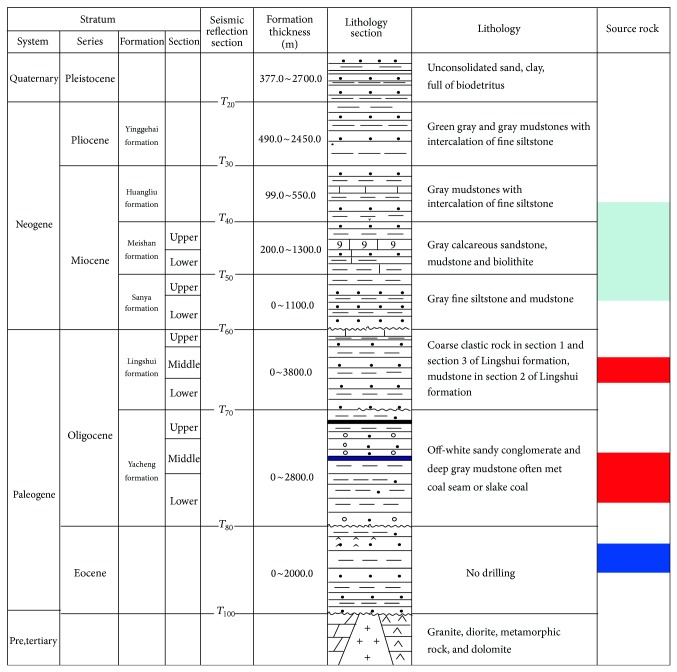
Comprehensive stratigraphic column of the Qiongdongnan Basin. Source rocks are marked with colors.

The basin is characterized by the double-layer structure. The lower structural layer, formed in the rifting stage, consists of the Eocene lacustrine deposition and the Oligocene including the Yacheng formation of transitional facies and the Lingshui formation of shallow sea deposits. Mudstones and coal seams developed in the Yacheng formation are the main source rocks in the basin (Figure 2). Marine dark grey mudstones in the Lingshui formation are considered to be the secondary important source rocks. In the early Oligocene (the sedimentary period of the Yacheng formation), the sedimentary environment was characterized by multiple uplifts and sags in the basin. OM supply was abundant enough to form high-quality source rocks. In the late Oligocene (the sedimentary period of the Lingshui formation), small-scale deltas were distributed in the basin. The supply of OM was also relatively abundant, which was favorable for the formation of source rocks. The upper structural layer formed in the depression period is comprised of Neogene and Quaternary, characterizing by marine strata. The overall structure in Miocene of the upper structural layer is characterized by weak deformation and little fault activities. During the sedimentary period of the Sanya formation, deposits of coastal, neritic, and bathyal facies were developed as a result of transgressive expansion. The northern continental slope, in which sediments of bathyal facies were developed, was also formed in that period. In the sedimentary period of the Meishan formation, the sedimentary range expanded further while the thickness of stratum decreased. Shallow platform was developed in the Yacheng salient zone, and fan delta was developed in the northern area of the basin. Neritic and bathyal facies, respectively, were formed in the middle uplift belt and the central depression belt. During the sedimentation of the Huangliu formation, a large-scale subsidence occurred and massive bathyal deposits were developed in the basin. Formations as Sanya, Meishan, and Huangliu in Miocene developed thick marine mudstones, which are the potential source rocks in the Qiongdongnan Basin (Figure 2). In Miocene, the supply of deposits was mainly from Hainan Island [12] rather than from the Red River, because the Red River is too far away from the Qiongdongnan Basin [10] and Hainan Island prevented sediments being transported from the Red River [13].

## 3. Data and Methodology

Trace element analysis is very useful for paleoproductivity and redox conditions [14–17]. Trace element data as well as maceral data were used to reconstruct depositional environment of the Miocene marine source rocks in the Qiongdongnan Basin. During the process, Al/Ti and P/Ti ratios were used to discuss paleoproductivity, and U/Th and Ni/Co ratios were used to recover the ancient redox conditions of the water column. The input of terrestrial higher plants was probed into based on maceral data. Seismic data were used to study the Miocene stratigraphy. Based on comparison of inorganic and organic geochemistry data, the depositional patterns of the Miocene marine source rocks were established, and the hydrocarbon potential of source rocks in different sedimentary environment was revealed. All the data (TOC and pyrolytic data, maceral data, and trace element data) were provided by China National Offshore Oil Corporation (CNOOC).

## 4. Results and Discussion

### 4.1. Hydrocarbon Potential of Miocene Marine Source Rocks

The TOC values in source rocks of Huangliu formation are ranging from 0.20% to 1.29%, with an average of 0.48% (Table 1). TOC values in the Meishan formation source rocks range from 0.18% to 1.81%, with an average of 0.45%. TOC values of Sanya formation range from 0.14% to 0.92%, with an average of 0.46%. Most samples from the study area were observed to reach the mature stage as their pyrolytic peak temperature (*T*_max⁡_) surpassed 430°C. The pyrolytic data such as hydrocarbon generating potential (*S*_1_ + *S*_2_) and hydrogen index (*I*_H_) are still on the low levels. All the above data from the exploratory wells indicate that Miocene marine source rocks have lower OM abundance and poor hydrocarbon potential in the Qiongdongnan Basin.

**Figure 3:**
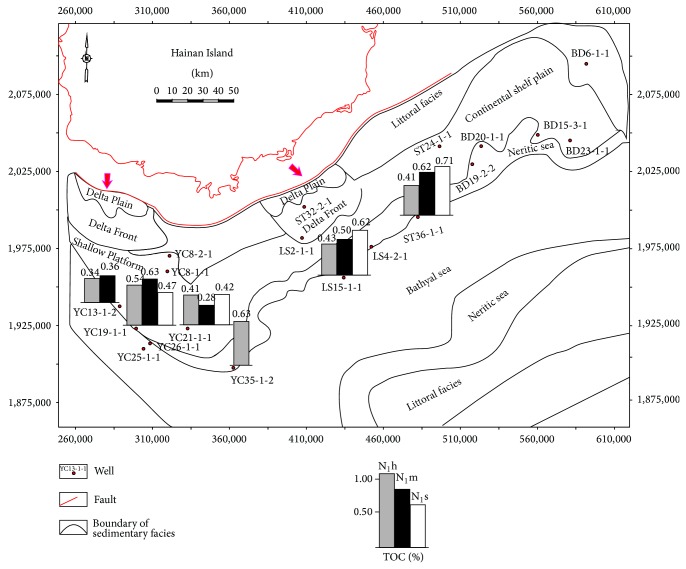
The distribution of TOC values of Miocene marine source rocks in some typical wells in the Qiongdongnan Basin.

The existing exploratory wells are mainly distributed in shallow water area, where the characteristics of source rocks are not representative for the whole area. However, from the shallow to relatively deeper water area, the TOC values of marine source rocks increase gradually. For example, data from three wells (YC35-1-2, LS15-1-1, and ST36-1-1) drilled in the margin of the central depression reveals an increasing trend of TOC of the Miocene marine source rocks from the shallow to relatively deeper water (Figure 3).

### 4.2. Palaeoenvironment in Which the Miocene Marine Source Rocks Formed 

#### 4.2.1. Input of TOM

Marine source rocks are not likely to be formed during the depression period. Basins with high-quality source rocks are often located under estuary of big rivers where TOM was plenty for forming source rocks. The Gulf of Mexico with Mississippi river and the lower Congo Basin with the Congo River system are two excellent examples. Oil and gas found in northern South China Sea were mainly generated from source rocks in transitional facies with terrestrial higher plants as the major supply. Petroleum from the Yacheng 13-1 gas field in the Qiongdongnan Basin mainly derived from the source rocks in Yacheng formation developed in transitional facies [18–22].

The TOM also played a significant role in the Baiyun Sag of the Pearl River Mouth Basin. The newly explored petroleum was mainly contributed by the source rocks in Enping formation developed in transitional facies, partly from marine source rocks in Zhuhai formation. Both aquatic organisms and terrigenous higher plants had contribution to the source rocks, but the latter played a more important role [23–25]. Available data also provide evidence for this conclusion. The data shows that the ancient Pearl River began to form since the sedimentary period of the Enping formation in the late Eocene. The influence of the river went so far that even source rocks in southern Baiyun Sag were affected during the sedimentary period of the Zhuhai formation. So the abundant TOM led to relatively high TOC values of source rocks in the Zhuhai formation.

**Figure 4:**
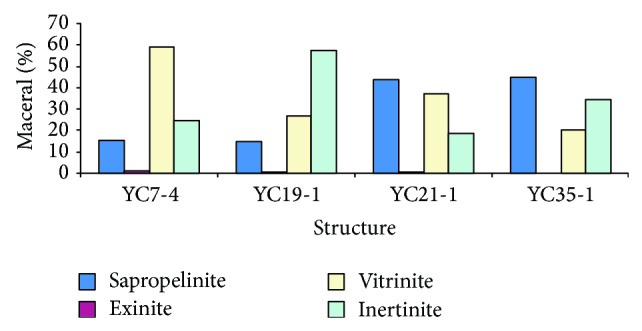
The maceral characteristics of the marine source rocks in the Sanya formation in different structures of the Qiongdongnan Basin.

However, due to the lack of large rivers or delta deposits, insufficient supply of TOM led to low OM abundance of the marine source rocks in the Lingshui, Sanya, and Meishan formations in the Qiongdongnan Basin. OM abundance of source rocks in the above formations is much lower than that from Zhuhai, Zhujiang, and Hanjiang formations, respectively, in Pearl River Mouth Basin [26]. To illustrate the idea, we chose maceral of the marine source rocks in the Sanya formation of the Qiongdongnan Basin as comparison basis to study the data from several exploratory wells drilled through structural belts from shallower water to deep water. The YC7-4, YC19-1, YC21-1, and YC35-1 structures were mainly developed in settings changing from fan delta and onshore to littoral-neritic and neritic to bathyal environment. The results show that, from shallower area to deep water, the marine source rocks in the Sanya formation have decreasing content of vitrinite and increasing content of sapropelinite (Figure 4), indicating lessening contribution of terrestrial higher plants and increasing input of algae to the formation of Miocene marine source rocks as water becomes deeper. In conclusion, the TOM input to Miocene marine source rocks in the Qiongdongnan Basin was relatively insufficient, and its contribution to the source rocks was shrinking with increasing water depth.

#### 4.2.2. Paleoproductivity

Calvert [27] suggested that high biologic productivity resulted in the high OM abundance of marine sedimentary rocks. Decomposition of abundant OM depletes oxygen dissolved in the water, providing favorable conditions for OM to accumulate on sea floor. Pedersen and Calvert [14] and Calvert et al. [28] believed that biological productivity in the water column was the most important factor controlling the formation of marine source rocks.

Previous studies provided us with a series of geochemical parameters for the estimation of paleoproductivity. Dymond et al. [29] and Francois et al. [30] suggested that the barium fluxes in sediments could be used to calculate the productivity. Paytan et al. [31] estimated the paleoproduction on the basis of measurements of BaSO_4_ concentration in sediments. Murray and Leinen [15] proposed that Al/Ti ratios could be used to evaluate the paleoproductivity. According to the sedimentary leaching results, more than 95% Ti was contained in refractory phases, and about 50% Al combined with the biogenic components [15]. Tyrrell [32] discussed the influence of nitrogen and phosphorus on the primary production and suggested that some creatures could get nitrogen from the air when NO_3_^−^/PO_4_^3−^ ratio was lower. But there is no alternative source once phosphate runs out. According to this view, the phosphate concentrations decide the nitrate concentrations, so phosphorus input controls the ocean primary production. Moreover, ^231^Pa/^230^Th [33], Ni [34, 35], and Cu [35] also can be used to evaluate paleoproductivity. Considering the limitations of each parameter, several proxies are synthesized to discuss the ocean primary production.

**Figure 5:**
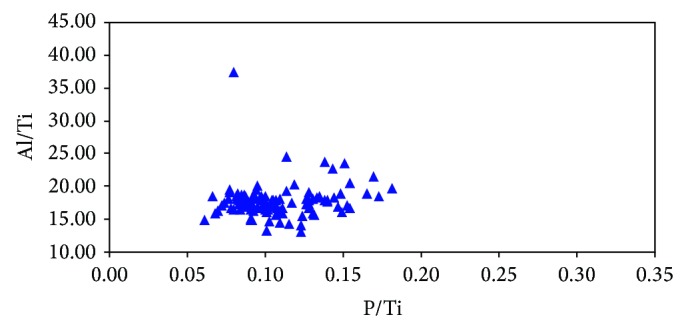
The cross plot showing the relationship between P/Ti ratios and Al/Ti ratios of Miocene source rocks in the Qiongdongnan Basin. Note: the samples in the chart are from the whole region of the basin. The information of the samples mainly represents the shallow water area because most of the wells distribute in the shallow water area (mainly in delta or coastal area).

**Figure 6:**
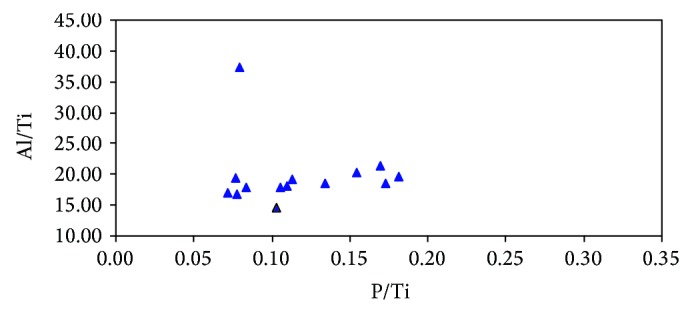
The cross plot showing the relationship between P/Ti ratios and Al/Ti ratios of Miocene source rocks of well BD20-1-1 in the Qiongdongnan Basin. Note: the samples in the chart are from the neritic sea.

Al/Ti and P/Ti are chosen in this paper. Al/Ti ratios of Miocene marine source rocks in the basin mainly distributes from 12.92 to 37.38 (Figure 5), with an average of 17.73, which is close to the average Al/Ti ratio (16.7) in Post-Archean Average Shale (PASS) [36], but is lower than those (35–41) associated with regions of high productivity in the modern equatorial Pacific [37]. The P/Ti ratios are in the range of 0.06 to 0.18 (Figure 5), with an average of 0.11, which approaches to the average P/Ti ratio (0.12) in PASS [36], but is lower than that (0.33) of average pelagic clay and far below those (2–8) in high productivity regions in the modern equatorial Pacific [37]. There is a poor correlation between P/Ti and Al/Ti ratios of the source rocks in the study area (Figure 5), because the exploratory wells in the Qiongdongnan Basin are mainly in the shallow water area where the Al/Ti ratio has been affected by terrestrial material input. The low Al/Ti and P/Ti ratios indicate low productivity. This can also be used to explain the fact of low OM abundance of the Miocene marine source rocks. However, the exploration well BD20-1-1 in relatively deep water at the edge of central depression belt reveals good linear relationship between P/Ti and Al/Ti of the source rocks (Figure 6). The average ratios of Al/Ti and P/Ti are 19.76 and 0.12, respectively (Table 2). Compared with the average ratios of P/Ti and Al/Ti in the study area, they are showing an increasing trend, indicating that the productivity turns better as the water depth increases from continental shelf to the edge of continental slope. It is therefore speculated that the productivity in bathyal continental slope (the central sag of the basin) was higher in Miocene.

#### 4.2.3. Redox Conditions

In the oxic environment, OM can be easily decomposed by dissolved oxygen in the water column and then the residual organic carbon can be consumed by benthic fauna on the seafloor. While in anoxic environment, OM is rarely consumed and may accumulate at the seafloor. Demaison and Moore [38] suggested that seawater with oxygen content less than 0.5 mL per liter could provide favorable preservation conditions for OM.

U/Th is usually used as an indicator for redox environment [39], and it is commonly high in organic-rich mudstones. Jones and Manning [40] concluded that U/Th ratio more than 0.75 indicated anoxic environment based on the fact that U/Th ratio decreases with rising oxygen concentration in water column. Tenger et al. [41] suggested that anoxic environment was possible when U/Th was more than 1.25 and Ni/Co ratio was over 7.00, oxic environment was implied when U/Th and Ni/Co ratios were less than 0.75 and 5.00, respectively, and a dysaerobic environment may be expected when U/Th ratio was ranging between 1.25 and 0.75 and Ni/Co ratio varies from 5.00 to 7.00. U and Mo are relatively stable in the oxygenated seawater, but compared with other metals, they are more easily to accumulate in anoxic environment, so U and Mo can be used as substitution index in anoxic environment [16, 42, 43]. Also, Mo mainly accumulates under the anoxic environment with sulfides. Adelson et al. [16] reconstructed the anoxic phase of Chesapeake Bay through the analysis of trace elements of Mo and Cu. Mn is absent in anoxic sediments but enriched in oxidized sediments, so it can be a reliable indicator under the oxygenated bottom water [42]. Other parameters, such as V/Ni, (Cu + Mo)/Zn, and V/Sc, can also be used to indicate redox conditions of bottom water as they increase with enhancing reduction in the water column [44, 45].

**Figure 7:**
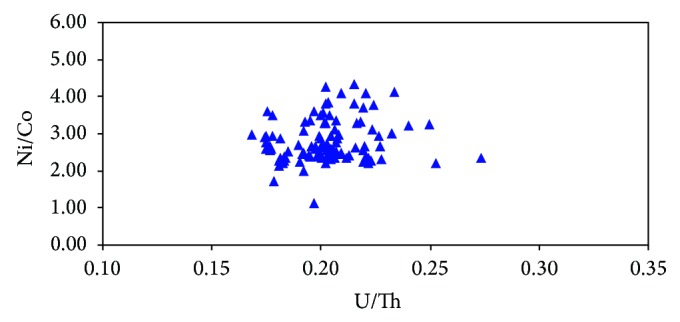
The cross plot showing the relationship between U/Th ratios and Ni/Co ratios of Miocene source rocks in the Qiongdongnan Basin. Note: the samples in the chart are from the whole region of the basin. The information of the samples mainly represents the shallow water area because most of the wells distribute in the shallow water area (mainly in delta or coastal area).

**Figure 8:**
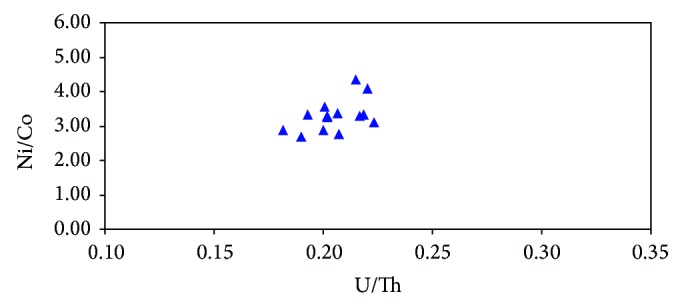
The cross plot showing the relationship between U/Th ratios and Ni/Co ratios of Miocene source rocks of well BD20-1-1 in the Qiongdongnan Basin. Note: the samples in the chart are from the neritic sea.

As shown in Figure 7, U/Th ratios of marine source rocks mainly range from 0.17 to 0.39 with an average of 0.20, and Ni/Co ratios vary from 1.13 to 4.32 with an average of 2.77. It infers that the source rocks were mainly developed in oxidized environment. The trace element from well BD20-1-1 near the central depression belt reveals that the average ratios of U/Th and Ni/Co of Miocene marine source rocks are 0.21 and 3.29, respectively (Table 2; Figure 8), which are relatively higher than the average ratios in marine source rocks mentioned above. The above analysis shows that Miocene source rocks were mainly developed in oxidized conditions, and the OM preservation conditions were getting better with water depth increasing.

### 4.3. The Depositional Patterns of Miocene Marine Source Rocks in the Qiongdongnan Basin

**Figure 9:**
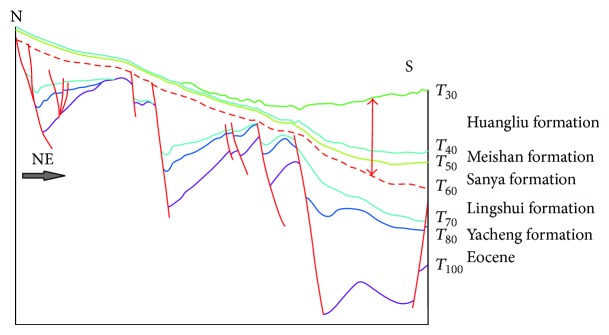
Seismic profile across Yabei, Yanan, and Ledong Sags in the west of the Qiongdongnan Basin. The position of the three sags is showed in Figure 1.

**Figure 10:**
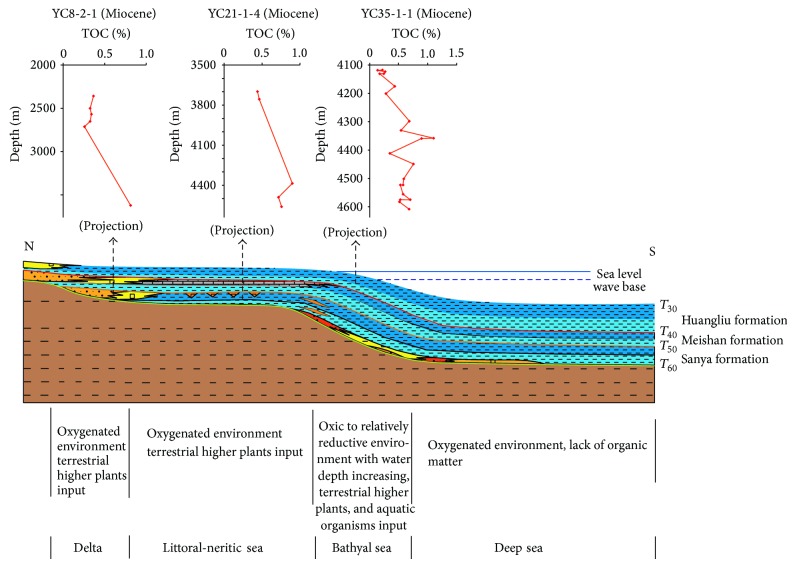
The depositional patterns of Miocene marine source rocks in the Qiongdongnan Basin.

Figure 9 shows a seismic section crossing three sags (Yabei, Yanan, and Ledong) in the west of the basin. The faulting activities controlled the development of the Oligocene stratum but were almost stopped during Miocene. The thickness of layers in Ledong Sag increased apparently in the central depression belt (Figure 9). Stratigraphic profile in Miocene is restored through the above section, which is shown in Figure 10. Data from wells YC8-2-1, YC21-1-4, and YC35-1-1 (the distance to the section is, resp., 5, 3, and 25 kilometers) reveal that the OM abundance of Miocene marine source rocks gets better with water depth increasing in the basin. It is attributed to the relatively better paleoproductivity and redox conditions from shallow area to deeper water though the contribution of TOM to source rocks was gradually decreasing. Thus it is speculated that the Miocene marine source rocks in a bathyal region, where there are still no drilling wells, have better hydrocarbon potential owing to relatively higher productivity and better preservation conditions of OM.

The depositional patterns of Miocene marine source rocks in the Qiongdongnan Basin can be summarized as follows (Figure 10). Source rocks in the continental shelf from north to south of the basin were less attractive in terms of hydrocarbon potential as they were mainly developed in delta and littoral-neritic sea environment, where terrestrial higher plants were the main OM contributor and the preservation conditions were unfavorable. Source rocks in the central depression belt of the continental slope have better hydrocarbon potential as they were formed in bathyal environment, where paleoproductivity was relatively higher and OM preservation conditions were better. Source rocks were not formed in pelagic sea environment, where OM was largely consumed in oxygenated water.

## 5. Conclusions

Terrestrial higher plants, paleoproductivity, and redox conditions are the main factors influencing the formation of Miocene marine sources rocks in the Qiongdongnan Basin. Source rocks were not developed in the shallow water area because of a shortage of TOM input and poor preservation conditions of OM. However, in the relatively deeper water region, the input of terrestrial higher plants decreased, with paleoproductivity and redox conditions playing a more important role in the formation of the Miocene marine source rocks.

Source rocks with better hydrocarbon potential are mainly distributed in a bathyal environment where the paleoproductivity was relatively high and preservation conditions were better, despite a sharply decreasing input of TOM. Source rocks in the continental shelf in delta and littoral-neritic environment have poor hydrocarbon potential because of unfavorable preservation conditions of OM.

## Figures and Tables

**Table 1:** Basic organic geochemical parameters for the Miocene marine source rocks in the Qiongdongnan Basin.

Formation	TOC, %			*S*_1_ + *S*_2_, mg/g			*I*_H_, mg/g			*T*_max⁡_, °C		
	Min.	Max.	Ave.	Min.	Max.	Ave.	Min.	Max.	Ave.	Min.	Max.	Ave.
N_1_h	0.20	1.29	0.48	0.07	2.44	0.43	11	332	52	406	468	434
N_1_m	0.18	1.81	0.45	0.12	6.23	0.69	22	331	80	401	465	436
N_1_s	0.14	0.92	0.46	0.12	2.59	0.45	20	361	61	402	482	448

Max., Min., Ave. represent maximum, minimum, and average data in the Table 1, respectively.

**Table 2:** Basic inorganic geochemical parameters for the Miocene marine source rocks of well BD20-1-1 in the Qiongdongnan Basin.

Depth (m)	Strata	Al/Ti	P/Ti	U/Th	Ni/Co
2290	N_1_m	14.68	0.10	0.21	4.32
2355	N_1_s	21.48	0.17	0.22	3.31
2395	N_1_s	20.38	0.15	0.22	4.08
2455	N_1_s	19.63	0.18	0.22	3.11
2515	N_1_s	18.51	0.17	0.22	3.27
2540	N_1_s	17.81	0.11	0.20	3.56
2570	N_1_s	19.26	0.11	0.19	3.32
2745	N_1_s	17.04	0.07	0.21	2.75
2775	N_1_s	16.72	0.08	0.20	3.26
2906	N_1_s	17.79	0.08	0.18	2.88
2964	N_1_s	18.43	0.13	0.19	2.67
2994	N_1_s	37.38	0.08	0.20	2.86
3050	N_1_s	19.48	0.08	0.20	3.28
3078	N_1_s	18.08	0.11	0.21	3.34
